# Effects of Multi-Muscle Electrical Stimulation and Stand Training on Stepping for an Individual With SCI

**DOI:** 10.3389/fnhum.2020.549965

**Published:** 2020-09-25

**Authors:** Kamyar Momeni, Arvind Ramanujam, Manikandan Ravi, Erica Garbarini, Gail F. Forrest

**Affiliations:** ^1^Tim and Caroline Reynolds Center for Spinal Stimulation, Kessler Foundation, West Orange, NJ, United States; ^2^Department of Physical Medicine and Rehabilitation, Rutgers New Jersey Medical School, Newark, NJ, United States

**Keywords:** spinal cord injury, multimuscle electrical stimulation, neuromuscular stimulation, stand training, locomotor training, body weight-supported training

## Abstract

The objective of this study was to evaluate the biomechanical, neural, and functional outcomes during a 10-min treadmill stepping trial before and after two independent interventions with neuromuscular electrical stimulation (ES) in an individual with spinal cord injury (SCI). In this longitudinal study, a 34-year-old male with sensory- and motor-complete SCI (C5/C6) underwent two consecutive interventions: 61 h of supine lower limb ES (ES-alone) followed by 51 h of ES combined with stand training (ST) using an overhead body-weight support (BWS) system (ST + ES). In post ES-alone (unloaded), compared to baseline, the majority (∼60%) of lower extremity muscles decreased their peak surface electromyography (sEMG) amplitude, while in post ST + ES (loaded), compared to post ES-alone, there was a restoration in muscle activation that endured the continuous 10-min stepping. Temporal α-motor neuron activity patterns were observed for the SCI participant. In post ST + ES, there were increases in spinal activity patterns during mid-stance at spinal levels L5–S2 for the right and left limbs. Moreover, in post ES-alone, trunk stability increased with excursions from the midline of the base-of-support (50%) to the left (44.2%; Baseline: 54.2%) and right (66.4%; baseline: 77.5%). The least amount of trunk excursion observed post ST + ES, from midline to left (43%; AB: 22%) and right (64%; AB: 64%). Overall, in post ES-alone, there were gains in trunk independence with a decrease in lower limb muscle activation, whereas in post ST + ES, there were gains in trunk independence and increased muscle activation in both bilateral trunk muscles as well as lower limb muscles during the treadmill stepping paradigm. The results of the study illustrate the importance of loading during the stimulation for neural and mechanical gains.

## Introduction

Motor-complete spinal cord injury (SCI) is associated with an inability to move below the level of a lesion resulting in skeletal unloading and rapid muscle atrophy (Spungen et al., 2003; Dudley-Javoroski and Shields, 2006; Momeni et al., 2019). The early musculoskeletal deterioration, while affecting both acute and chronic health conditions, can directly impact future functional mobility, trunk independence, and independent standing or walking (Spungen et al., 2003; Gorgey and Dudley, 2007; Gorgey et al., 2012).

The combination of step and stand training protocols, such as locomotor training, and stand training (ST) alone have been extensively published to show improvements in seated balance, independent standing, gains in stepping and walking measures for individuals with a motor-incomplete SCI (Forrest et al., 2008b; Forrest et al., 2008a, 2012; Harkema et al., 2011, 2012b; Buehner et al., 2012). However, for individuals with complete lesions, these activity-based interventions do not seem to be sufficient for functional gains in the lower extremity (Qin et al., 2010), although several published studies do show the influence of activity-based therapies for positive lower extremity neurophysiological or neuromuscular modulations after having completed a large number of training sessions (Forrest et al., 2008b; Mitchell et al., 2015; Canton et al., 2016).

Previous studies on longitudinal applications of neuromuscular electrical stimulation (ES) for a single- or multimuscle-elicited contractions have shown a direct improvement in skeletal muscle size and strength, as well as body composition, metabolic profile, function, neuromuscular control, and reduction of fatigue in stimulated muscles (Mahoney et al., 2005; Sabatier et al., 2006; Gorgey et al., 2012, 2015; Ryan et al., 2013) for both chronic and acute SCI. Moreover, multimuscle ES of the lower and upper extremity have shown an improvement in muscle function even during activities of daily living (Popovic et al., 2006; Kapadia et al., 2014). When lower limb multimuscle ES is combined with ST and compared to lower limb multimuscle ES alone (without loading), there are increased gains in lower limb muscle hypertrophy (Forrest G. et al., 2009), trunk control, and trunk muscle activation during independent standing leading to independent standing postural balance. In the present investigation, we extend our earlier work (Momeni et al., 2019) to identify and characterize alterations in the neuromuscular, biomechanical, and functional adaptations during a treadmill stepping paradigm after ST combined with lower limb multi-muscle ES. Also, we will demonstrate the activity-dependent plasticity associated with loading during multimuscle ES compared to multimuscle ES alone (without loading). Loaded vs. unloaded training conditions accompanied the improved locomotion and dynamic posture, as observed in the individual with motor-complete SCI.

## Methods

An individual with chronic, motor-complete SCI (neurological level of injury at C5/C6) and an able-bodied (AB) individual participated in this study (Table 1). Neuromuscular and biomechanical assessments were completed for the participant with SCI: (1) prior to any intervention, (2) after 61 h of bilateral, lower limb ES in supine (ES-alone), and (3) after 51 h of dynamic ST combined with bilateral, lower limb ES (ST + ES). Similar biomechanical and neuromuscular assessments were also completed for AB at one time point, without any intervention.

**TABLE 1:** Demographics of the participant with spinal cord injury (SCI) and the able-body control (AB).

**Participant**	**Age (Year)**	**Height (cm)**	**Weight (kg)**	**TSI^a^ (Months)**	**AIS^b^**	**Intervention (*h*)**
SCI	34	180.3	66.4	19	A	61 (ES-alone)
						51 (ST + ES)
AB	25	185.0	76.4	–	–	–

*^*a*^Time since injury. ^*b*^American Spinal Injury Association Impairment Scale from The International Standards for Neurological Classification of Spinal Cord Injury (ISNCSCI) (Marino et al., 2008; Schuld et al., 2016).*

Training sessions occurred three to four times per week, each for a duration of 1 h (Table 1). All procedures were approved by the Kessler Foundation’s Institutional Review Board. Informed consent was obtained before participation.

### Training Protocol

#### Supine Electrical Stimulation (ES-Alone)

Neuromuscular electrical stimulation (ES) was applied in the supine position via bifurcated leads and self-adhesive reusable surface electrodes. Self-adhesive stimulation electrodes were placed over the motor points of bilateral upper-leg and lower-leg muscles: quadriceps muscle group (QUAD), hamstrings muscle group (HAM), gastrocnemius (GN), and tibialis anterior (TA). Two 5 × 10-cm oval electrodes were placed on each of the RF and BF muscles and two 5 × 5-cm square electrodes on each of the GN and TA muscles. Biphasic, square-wave electrical pulses were applied, using the Rehabilicare IF 3WAVE System (Compex Technologies Inc., New Brighton, MN, United States). Symmetrical 300-μs biphasic, square-wave pulses at 35 Hz were delivered over a duty cycle of 11 s on, 60 s off. Stimulation intensity was determined for each muscle and periodically adjusted throughout the interventions, based on participant’s muscle response with a maximum amplitude of 100 mA. During the 11-s stimulation window in each cycle, stimulation pulses were applied to the lower-leg muscles first; then after 4 s, it was also applied to the upper-leg muscles for the remaining 7 s, creating a 7-s stimulation overlap between the upper- and lower-leg muscles in each cycle (Momeni et al., 2019).

Prior to training, the participant was acclimated to electrical stimulation during the process of determining the highest tolerable level of ES that produced both visible and palpable contractions in all muscles; these values were then recorded to be used and adjusted throughout the training.

#### Stand Training and Electrical Stimulation (ST + ES)

This is a combination of the multimuscle ES, mentioned above, and stand training, which involves a series of standardized dynamic tasks while standing using an overhead body-weight support (BWS) system (Robomedica^®^, Irvine, CA, United States). Training was divided into two different modes of stand training: stand adaptability (at higher percentage of BWS) and stand retraining (at lower percentage of BWS) (Harkema et al., 2012a). Stand adaptability training at higher percentage of BWS provided greater assistance with increased body weight assistance for an individual to complete the different stand training tasks independently with less assistance from trainers. Stand retraining was performed with the minimum BWS (0%, if possible), with the trainers providing assistance during the stand training tasks, promoting weight bearing during standing. In addition to quiet standing as a task, other tasks were performed, such as lateral and anterior weight shifts, etc.

Further details of the stand training protocol are described in our previous work (Momeni et al., 2019). Once every 10 min throughout the training, the heart rate and blood pressure were monitored for the safety of the participant (Canton et al., 2016; Momeni et al., 2016).

### Data Collection

Data were collected at baseline, post ES-alone intervention, and post ST + ES intervention. The participant performed a 10-min assisted-stepping session on a treadmill with a speed of 0.8 m/s and 60% BWS, but without use of ES; manual assistance at pelvis and bilateral knees was provided by trained therapists (Behrman et al., 2005). The AB participant performed two walking trials on the BWS treadmill: (1) with harness and 50% BWS and (2) without harness, both at a speed of 1.5 m/s to elicit bilateral muscle activations synonymous with walking (Anders et al., 2007).

#### Neuromuscular Data

Surface electromyography (sEMG) data were collected at 2,520 Hz using two EMG systems (MA-100 and MA-300, Motion Lab Systems Inc., Baton Rouge, LA, United States) and stainless steel, differential input design, and surface electrodes with an inter-electrode distance of 18 mm (Motion Lab Systems Inc., Baton Rouge, LA, United States). EMG electrodes were placed bilaterally on the following muscles: erector spinae T5 (SES), erector spinae T12 (IES), external obliques (EO), internal obliques (IO), gluteus maximus (GM), rectus femoris (RF), vastus lateralis (VL), biceps femoris (BF), tibialis anterior (TA), gastrocnemius (GN), and soleus (S). Electrode placement protocol has been explained in further detail elsewhere (Kendall et al., 2013; Momeni et al., 2019). Reference electrodes were placed on the clavicles.

#### Biomechanical/Kinematics Data

Kinematic data were collected at 60 Hz using a motion capture system (Vicon Motion Systems Ltd., Oxford Metrics, United Kingdom). Reflective markers were placed on specific anatomical landmarks according to the Vicon Plug-in Gait marker set.

### Data Analysis

The first-, fifth-, and tenth-minute periods of the 10-min assisted-stepping trials were extracted for further analysis and are referred to as time periods (t_1_, t_5_, and t_10_, respectively). Kinematic data were filtered using a low-pass (cut-off: 6 Hz) Butterworth filter (fourth order, zero lag). The bilateral heel and toe markers’ 3-D coordinates were used to determine the instantaneous base of support (*BoS*), defined as the distance between the left and right heel markers, and gait cycle events (i.e., Heel strike and Toe off) for the stepping trials. Gait cycles were determined between consecutive ipsilateral heel strikes and time normalized. Trunk model (de Leva, 1996), using acromion and anterior superior iliac spine (ASIS) markers, determined: (i) sagittal plane excursion for trunk center-of-mass (*CoM*_T,AP_), relative to left heel marker, normalized to mean step length (defined as the anterior–posterior distance between the bilateral heel markers at consecutive heel strikes), and duration of each gait cycle (${\hat{C\,o\,M}}_{T,\mathrm{AP}}$) and ii) frontal plane excursion for trunk center-of-mass (*CoM*_T,ML_), relative to left heel marker, normalized to the instantaneous width of BoS and duration of each gait cycle (${\hat{\text{CoM}}}_{T,\mathrm{ML}}$). Profiles were then averaged for the first minute of the stepping trial.

Quantification of sEMG was completed through custom-written programs, developed in MATLAB (MathWorks^TM^, Natick, MA, United States). Surface EMG data were gain-normalized, full-wave rectified, and filtered using band-pass (20–150 Hz) and band-stop (60 ± 3 Hz) Butterworth filters (fourth order, zero lag) for further analysis. The onset and cessation of EMG bursts during each gait cycle were defined using the Teager–Kaiser energy operator (TKEO) as previously described (Solnik et al., 2010; Pilkar et al., 2012; Canton et al., 2016; Momeni et al., 2016). The TKEO measures instantaneous energy changes of a signal and amplifies the energy of the action potential spikes; therefore, it differentiates between the relaxed and contracted states of the muscle. Further, the TKEO output is derived from the instantaneous amplitude and frequency of the signal; hence, the TKEO conditioning flattens the low-frequency baseline during non-active periods, reduces false onset detection, and increases robustness of computerized methods (Solnik et al., 2010). To identify the onset and cessation of sEMG activation, the TKEO output for each muscle was used to calculate the baseline noise and establish a detection threshold of seven standard deviations above the calculated baseline.

Mean and peak burst amplitude (μV), and burst duration as a percentage of gait cycle (%GC) were calculated for each muscle. EMG variables were calculated for each gait cycle separately at three time periods, first-, fifth-, and tenth-minute (t_1_, t_5_, t_10_), during the 10-min stepping trial at baseline, post ES-alone, and post ST + ES. Therefore, each mean EMG measure had three values at baseline, three at post ES-alone, and three at post ST + ES (Table 2).

**TABLE 2:** Burst duration, as a percentage of gait cycle (%GC), and mean EMG (μV) values for the left limb of the SCI participant.

	**Muscle**	**BASELINE**			**ES-alone**			**ST + ES**		
		**1st min**	**5th min**	**10th min**	**1st min**	**5th min**	**10th min**	**1st min**	**5th min**	**10th min**
BURST DURATION (%GC)	GM	127	136	106	53	63	84	97	176	147
	BF	4211	299	4212	3410	2617	3411	347	258	126
	RF	709	629	5214	409	5011	369	287	317	298
	VL	6211	397	3012	289	2911	157	337	317	267
	TA	6015	409	4613	579	4510	3610	428	4211	2010
	GN	747	647	457	148	2210	2510	607	6811	518
	S	648	587	669	8211	8510	6310	486	6814	436
	SES	5517	7419	7415	6518	6017	7619	5818	5514	7022
	IES	3214	7713	7313	8510	7116	9012	6913	8311	7513
	EO	4317	6220	7518	7316	6721	9114	2311	167	157
	IO	4917	7422	7416	5119	5218	5423	7419	6110	6319
MEAN EMG (μV)	GM	0.700.06	0.620.05	0.610.04	0.760.04	0.780.04	0.760.04	0.780.06	0.710.04	0.730.05
	BF	1.240.14	1.020.11	0.890.08	1.250.07	1.640.20	1.380.14	1.120.16	0.880.08	0.820.05
	RF	3.350.49	2.680.46	2.460.52	1.880.29	2.020.35	1.930.31	1.150.13	1.470.29	1.360.19
	VL	2.970.59	1.790.43	1.220.28	0.180.03	0.170.03	0.160.02	1.760.27	1.780.40	1.180.16
	TA	2.170.53	1.490.13	1.160.11	1.930.34	1.270.17	1.150.11	2.120.28	1.600.22	1.360.13
	GN	5.830.76	4.090.33	3.350.26	4.970.42	3.290.30	3.240.21	4.100.52	2.670.29	2.090.18
	S	6.520.54	5.830.46	5.200.78	4.540.94	3.710.68	4.020.44	7.750.79	7.071.12	6.380.59
	SES	3.260.32	2.990.60	2.260.36	37.726.48	26.915.24	1.360.29	2.020.32	1.610.23	5.111.53
	IES	24.072.36	10.861.60	9.661.68	17.722.37	14.772.69	17.874.75	36.545.34	31.565.42	30.645.12
	EO	4.970.65	6.481.33	6.051.09	3.000.40	3.170.58	4.191.01	1.770.21	1.680.19	1.790.22
	IO	3.400.34	3.020.61	2.290.37	3.260.47	2.800.48	1.410.28	25.494.10	4.400.45	3.050.88

Three measures of muscle co-contraction were calculated: co-excitation (CE), co-inhibition (CI), and co-activation (CA). CE was defined as the time period that two muscles were simultaneously active, relative to the total time either muscle was active during each gait cycle. CI was defined as the time period that two muscles were simultaneously inactive, relative to the total time either muscle was inactive, during each gait cycle. CA was defined as the mean of CE and CI, within each gait cycle. Values were: (i) averaged over multiple gait cycles for each given time period (t_1_, t_5_, and t_10_) and (ii) presented as percentages of the gait cycle. An index value of 100% indicates complete CA, CE, or CI of activation, whereas a value of 0% indicates no CA, CE, or CI of activation (Johnson, 2003; Canton et al., 2016; Momeni et al., 2016).

For lower limb muscles, co-contraction (CA, CE, and CI) values were determined for the ipsilateral agonist/antagonist muscle pairings (i.e., RF/BF, RF/GM, VL/GM, VL/BF, TA/GN, and TA/S) whereas for trunk muscles, co-contraction values were determined for the ipsilateral trunk muscle pairings (i.e., SES/IES and IO/EO). Each of the CA, CE, and CI values was calculated at t_1_, t_5_, and t_10_ periods of baseline, post ES-alone, and post ST + ES. Therefore, each muscle co-contraction pair had three values at baseline, three at post ES-alone, and three at post ST + ES. For instance, for the two shank muscle pairs (TA/GN and TA/S), there is a total of 12 indices available bilaterally for all time periods combined (i.e., 2 muscle pairs × 2 limbs × 3 time periods).

Statistical analyses were performed using IBM SPSS (v.26, IBM Corp., Armonk, NY, United States). Descriptive statistics include mean ± standard deviation. To compare each of these outcome measures derived from individual gait cycles (total of ∼30–40 gait cycles) for each time period, multiple paired *T*-tests were performed determining significant differences between consecutive time points (i.e., post ES-alone and baseline, post ST + ES and post ES-alone). Significance level was set at 0.05.

We generated spatiotemporal maps of the α-motorneuron (MN) activities during assisted stepping to distinguish the approximate location of ipsilateral MN pools in the rostrocaudal axis of the human spinal cord and to further determine the characteristics of the locomotion circuitry. EMG and kinematics data were used to construct maps of spinal MN activity according to myotomal charts of Kendall et al. (2005). These spinal localization charts were created based on anatomical and clinical data (Kendall et al., 2005); thus, it is assumed that our participants have the same spinal topography as the reference population. The recorded muscle activity patterns were weighed and mapped onto the approximate location of the ipsilateral spinal MN pools (Ivanenko et al., 2006). For each spinal segment, *S*_j_, contributions of any number of rectified EMGs corresponding to that segment were weighed, separately, and then averaged:

$$S_{j}=\frac{\sum_{i=1}^{n_{j}}w_{i\,j}.E\,M\,G_{i}}{n_{j}}\;\left(1\right)$$

where *n*_*j*_ is the total number of EMG signals corresponding to the *j*th spinal segment, and *w*_*ij*_ is the weighing coefficient for the *i*th muscle, within the *j*th spinal segment. Weighing coefficients are based on Kendall et al. (2005) reference segmental charts for all muscles, compiled by combining anatomical and clinical data from six sources; they assigned “X” to localizations agreed on by five or more sources and “x” to those agreed on by three to four sources. We have utilized Ivanenko’s method (Ivanenko et al., 2006) that assigns a weighing coefficient of 1 and 0.5 to “X” and “x,” respectively.

A total of 24 activation waveforms were derived based on the 24 anatomical vertebrate segments covering spinal levels C4 through S2, corresponding to the levels at which the motorneurons innervate the recorded muscles. Note – data were recorded from 22 bilateral muscles with the assumption that rectified EMG waveforms, utilized for generating maps, provide an indirect measure of each muscle’s α-motorneurons’ net activity in the spinal cord. To generate the smoothed spatiotemporal maps, filled contour plots were created from the mean of the activation waveform matrix across multiple time-normalized gait cycles, separated by stance and swing phases.

## Results

**FIGURE 1:**
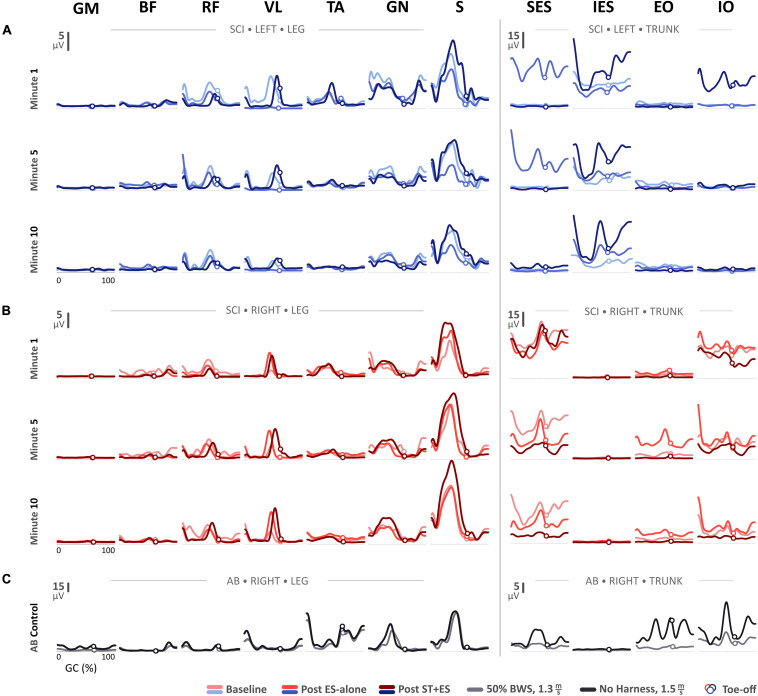
Mean electromyography (EMG) profiles for **(A)** the SCI participant’s left side and **(B)** right side during the first-, fifth-, and tenth-minute of the assisted-stepping trials at baseline, post ES-alone, and post ST + ES. **(C)** Mean EMG profiles for the able-bodied (AB) participant’s right side during walking on a treadmill at 50% body-weight support and without harness or any body-weight support.

For the SCI participant, the linear envelope of the mean rectified sEMG profiles for t_1_, t_5_, and t_10_ at baseline, post ES-alone, and post ST + ES are shown in Figure 1. Profiles for the AB control during independent treadmill stepping are also shown in Figure 1.

### Lower Extremity Muscle Activations

#### Mean Amplitude

In post ES-alone, compared to baseline measures, the mean amplitude (Figure 1) for bilateral S muscles decreased for t_1_, t_5_, and t_10_ (*p* < 0.000 bilaterally), except for right S muscle at t_1_. In post ST + ES, compared to post ES-alone, the mean amplitudes for S muscles increased bilaterally in all time periods (*p* < 0.000 bilaterally).

#### Peak Amplitude

**FIGURE 2:**
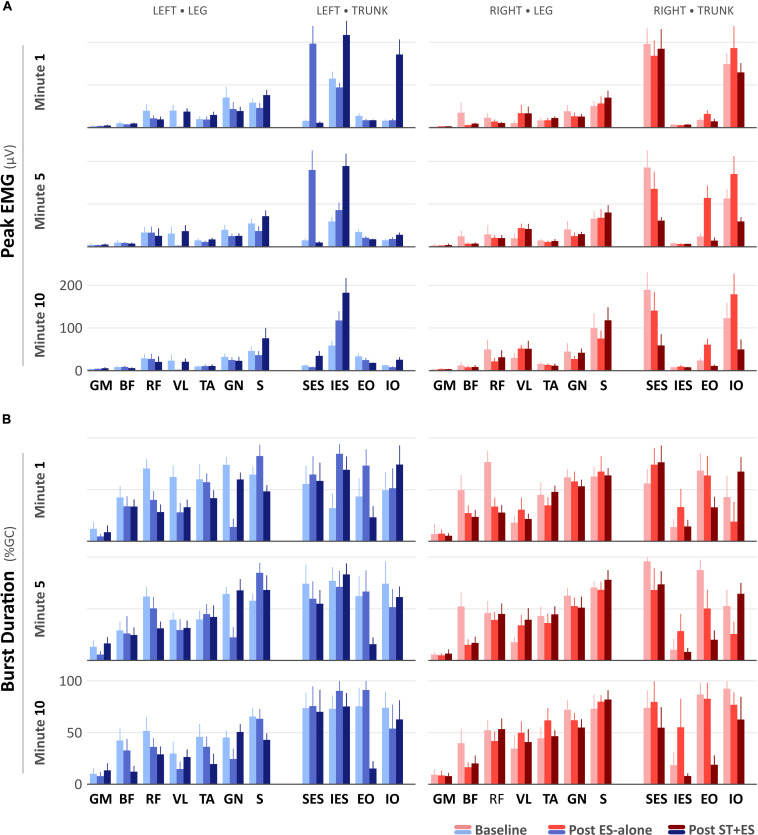
**(A)** Peak electromyography (μV) and **(B)** burst duration (% Gait Cycle) values for the SCI participant during the first-, fifth-, and tenth-minute of the assisted-stepping trials at baseline, post ES-alone, and post ST + ES.

For all time periods (t_1_, t_5_, and t_10_), peak EMG amplitudes (Figure 2A) for left RF (*p* < 0.000), VL (*p* < 0.000), GN (p < 0.000), and S (*p* < 0.000), and right RF (*p* < 0.000), BF (*p* < 0.000), TA (*p* = 0.007), GN (*p* < 0.000, except for t_10_) decreased post ES-alone, compared to baseline. In post ST + ES, compared to ES-alone, peak EMG amplitudes for left VL (*p* < 0.000), TA (*p* < 0.000), S (*p* < 0.000) and for right BF (*p* < 0.000), TA (*p* = 0.018), S (*p* < 0.000), GN (*p* < 0.000) increased for all time periods.

#### Burst Duration

In post ES-alone, compared to baseline, burst duration (Figure 2B) decreased bilaterally for RF (*p* < 0.000), BF (*p* < 0.000), GN (*p* < 0.000) muscles, and left VL (*p* < 0.000) for all time periods, while for bilateral S (*p* < 0.014) muscles, it decreased in two of six time periods (i.e., 2/6). In post ST + ES, compared to ES-alone, burst duration increased in seven different muscles, including left GM (*p* < 0.000), VL (*p* < 0.000), GN (*p* < 0.000), and right BF (*p* = 0.036), RF (*p* < 0.000), TA (*p* < 0.000), S (*p* < 0.000) in two of three time periods, while it decreased for left RF (*p* < 0.000), BF (*p* = 0.001), TA (*p* < 0.000), and S (*p* < 0.000) in all time periods (t_1_, t_5_, and t_10_).

### Trunk Muscle Activations

#### Mean Amplitude

In post ES-alone, compared to baseline, for all time periods (t_1_, t_5_, t_10_), the mean amplitude (Figure 1) for left SES (*p* < 0.000), IES (*p* < 0.000), and right IO (*p* < 0.000) increased, while it decreased for right SES (*p* < 0.000). In post ST + ES, compared to ES-alone, the mean amplitude for left IES (*p* < 0.000) and IO (*p* < 0.000) increased for t_1_, t_5_, and t_10_, however, it decreased for right IO (*p* < 0.000). Bilateral SES (*p* < 0.000) amplitude also decreased in five of six time periods.

#### Peak Amplitude

In Post ES-alone, the peak EMG amplitude increased for left SES (*p* < 0.000), IES (*p* < 0.000), and right EO (*p* < 0.000), IO (*p* < 0.000); left EO (*p* < 0.000) increased in 11 of 12 time periods. Right SES (*p* < 0.000) peak EMG amplitude decreased for t_1_, t_5_, and t_10_. In post ST + ES, compared to ES-alone, the peak EMG amplitude increased for left IES (*p* < 0.000) and IO (*p* < 0.000) for t_1_, t_5_, and t_10_. Bilateral SES (*p* < 0.000) and right IO (*p* < 0.000) and EO (*p* < 0.000) decreased the peak EMG amplitude in 14 of 15 time periods (Figure 2A).

#### Burst Duration

In Post ES-alone, burst duration (Figure 2B) increased for bilateral IES (*p* < 0.000) and left EO (*p* < 0.000) in 10 of 12 time periods. In post ST + ES, compared to post ES-alone, burst duration increased for bilateral IO (*p* < 0.000, except the right IO at t_10_) at each time period (t_1_, t_5_, and t_10_). Burst duration decreased in all other muscles for t_1_, t_5_, and t_10_.

### Lower Extremity Muscle Co-contraction

**FIGURE 3:**
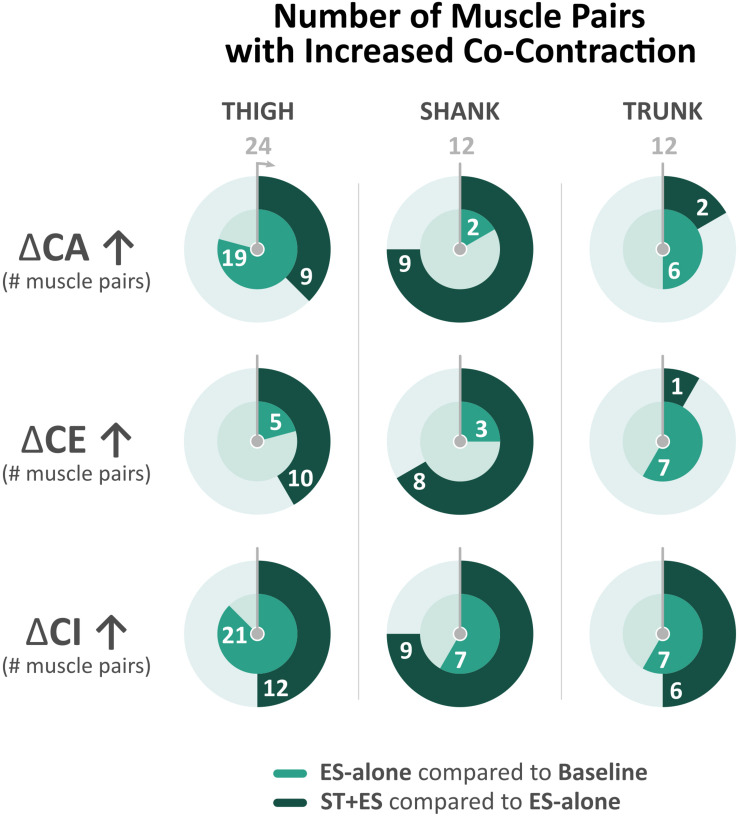
Number of bilateral muscle pairs with a numerical increase in co-contraction measures (i.e., co-activation, co-excitation, and co-inhibition) across all time periods (t_1_, t_5_, and t_10_) for electrical stimulation (ES)-alone relative to baseline and stand training (ST) + ES relative to ES-alone. Total number of muscle pairs across all time periods is 24 for the thigh, 12 for the shank, and 12 for the trunk.

In post ES-alone, 19 of 24 bilateral thigh agonist/antagonist CA indices increased significantly (*p* < 0.015) for all time periods (t_1_, t_5_, and t_10_). CA indices for left VL/BF (*p* < 0.015, except at t_5_) and right VL/GM (*p* < 0.000, except at t_1_) significantly decreased. Bilateral shank (TA/GN, TA/S) agonist/antagonist CA decreased in 10 of 12 calculated indices (*p*_Left TA/GN_ < 0.009 except at t_10_, *p*_Right TA/__GN_ = 0.014 only at t_1_, *p*_Left TA/S_ < 0.000 except at t_10_, and *p*_Right TA/S_ = 0.002 only at t_5_). In post ES-alone, 18 CE indices for left thigh and shank decreased significantly (*p* < 0.024). Nineteen (19 of 24) bilateral thigh and three (3 of 12) shank CI indices increased significantly (*p* < 0.006 and *p* < 0.004, respectively); three of 6 TA/S CI indices decreased (*p* < 0.011) (Figure 3).

In post ST + ES, 11 of 24 bilateral thigh CA indices decreased significantly over all time periods (*p* < 0.029). Bilateral shank muscle agonist/antagonist CA increased significantly in 9 of 12 calculated indices (*p* < 0.014). Seven (7 of 24) bilateral thigh CE indices decreased significantly (*p* < 0.044), while 8 of 12 shank CE indices increased (*p* < 0.044). Bilateral CI increased significantly in 9 of 24 thigh indices (*p* < 0.029) as well as 8 of 12 shank indices (*p* < 0.043), which includes significant increases in four of six bilateral TA/S CI indices (*p* < 0.000) (Figure 3).

### Trunk Muscle Co-contraction

In post ES-alone, 6 of 12 CA indices increased bilaterally across all time periods (t_1_, t_5_, and t_10_) (*p* < 0.004), while CE increased significantly in 6 of 12 (*p* < 0.006) and CI increased in 6 of 12 calculated indices (*p* < 0.000) (Figure 3). In post ST + ES, compared to ES-alone, 2 of 12 CA indices increased significantly (*p* < 0.017), while CE increased in 1 of 12 (*p* < 0.000) and CI increased in 6 of 12 calculated indices (*p* < 0.015) with four of six occurring in the t_10_ time period.

### TrunkCenter of Mass

**FIGURE 4:**
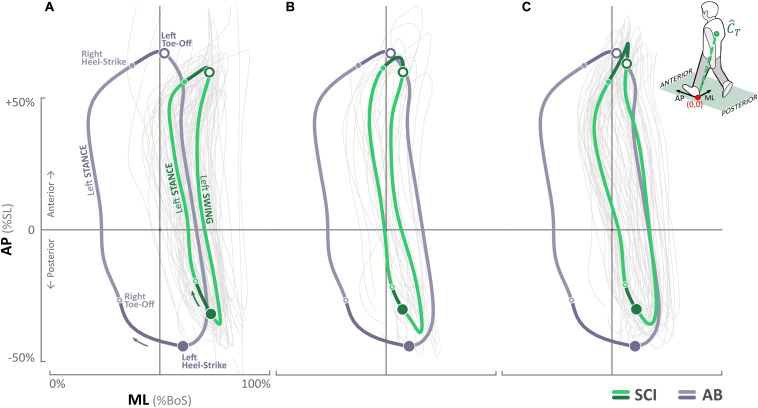
Trunk center of mass (${\hat{C\,o\,M}}_{\text{T}}$) trajectories for the SCI participant in the anterior–posterior (AP) and medial–lateral (ML) directions at **(A)** baseline, **(B)** post ES-alone, and **(C)** post ST + ES. Trunk center of mass (${\hat{C\,o\,M}}_{\text{T}}$) trajectories of the AB participant are overlaid for comparison. The direction of the movement and locations of right and left heel strikes and toe offs are marked in **(A)**.

For AB control, the ${\hat{C\,o\,M}}_{\text{T}}$ trajectories are shown in Figure 4. ${\hat{C\,o\,M}}_{T,\mathrm{ML}}$ excursion during stepping ranged from 14.6 to 74.2% of *BoS* with left and right mid-stance occurring mediolaterally at 22.05 and 63.98%, respectively, about the midpoint of *BoS* (i.e., 50%). Mean step length ($\bar{S\,L}$) was 75.97 ± 0.96 cm, and mean BoS ($\bar{B\,o\,S}$) was 9.65 ± 1.47 cm throughout the trial.

For the SCI participant, baseline ${\hat{C\,o\,M}}_{T,\mathrm{ML}}$ trajectories ranged from 54.2 to 77.5% *BoS* with left and right mid-stance at 56.32 and 68.01%, respectively (Figure 4); post ES-alone, ${\hat{C\,o\,M}}_{T,\mathrm{ML}}$ excursion increased significantly (*p* < 0.000) and ranged from 42.2 to 66.4% of *BoS* with left and right mid-stance at 42.90 and 54.60%, respectively; in post ST + ES, ${\hat{C\,o\,M}}_{T,\mathrm{ML}}$ further increased (significant increase on the right side, *p* < 0.000) and ranged from 41.9 to 70.2% of *BoS* with left and right mid-stance at 42.64 and 64.16%, respectively. In post ES-alone, $\bar{S\,L}$ decreased from 57.21 ± 5.43 cm (baseline) to 51.78 ± 3.47 cm, and in post ST + ES, it increased to 61.55 ± 3.43 cm. Changes in $\bar{B\,o\,S}$ at post ES-alone, compared to baseline, were minimal from 23.97 ± 2.05 cm to 23.78 ± 2.57 cm, whereas in post ST + ES, there was an increase to 26.24 ± 3.14 cm. Note – these $\bar{S\,L}$ and $\bar{B\,o\,S}$ values are less than the AB values. The ${\hat{C\,o\,M}}_{T,\mathrm{AP}}$ ranges at baseline, post ES-alone, and post ST + ES were −35.8 to 61.6%, −38.9 to 65.5%, and −35.8 to 70.7% of SL, respectively, and similar to AB control ${\hat{C\,o\,M}}_{T,\mathrm{AP}}$ trajectories, which ranged from −42.7 to 66.0% of SL.

### Spinal Motorneuron Activity Patterns

**FIGURE 5:**
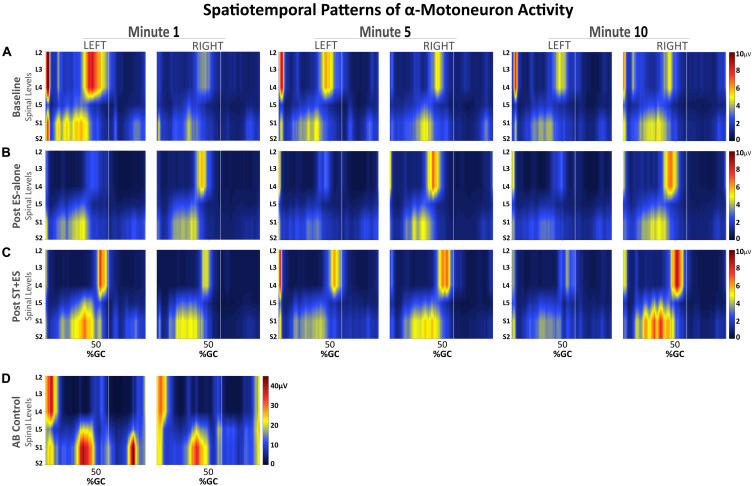
Spatiotemporal patterns of α-motorneuron activation along the rostrocaudal axis of the spinal cord for the SCI participant **(A–C)** during assisted stepping on a treadmill with overhead body-weight support and for the able-bodied participant **(D)** during walking on a treadmill. Each segment on the vertical axis was reconstructed based on the recorded, non-normalized EMG signals from segmental localization charts (Kendall et al., 2005). Horizontal axis represents the time-normalized gait cycle with a vertical line that marks where the toe off occurs.

We used the recorded EMG data from 22 bilateral muscles to construct the spinal MN activity maps during assisted stepping for all time periods at baseline, post ES-alone, and post ST + ES. Figure 5 illustrates the resulting maps of lumbosacral MN activity during *t*_*1*_ of assisted stepping at baseline and post ES-alone and ST + ES interventions.

Observed bursts in the AB activity maps (Figure 5D) are consistent with previously published data on able-bodied individuals walking overground, with the exception of activity during mid-swing at S1-2 levels (Ivanenko et al., 2006). Temporal activation patterns are evident in all MN activity maps for the SCI participant (Figures 5A–C), however, there are apparent differences before and after interventions, compared to AB, especially during mid-swing at spinal levels L5–S2. Activation patterns for the SCI participant did show within-subject similarities, although in post ST + ES intervention, there were increases in spinal activation patterns during mid-stance at spinal levels L5–S2 for t_1_, t_5_, and t_10_ for the right limb and t_1_ for the left limb, as noted for the AB participant.

## Discussion

In the current study, for an individual with SCI, we characterized the biomechanical, neural, and functional effects for longitudinal, multimuscle ES during dynamic loading (ST + ES) compared to unloaded (ES-alone) conditions during 10 min of continuous treadmill stepping. Data from the first, fifth, and tenth minutes (t_1_, t_5_, and t_10_) of the 10-min condition identified the impact of the neural afferents associated with loading could influence locomotor outcome for muscle amplitude even after longitudinal stand training.

In post unloaded condition (ES-alone), compared to baseline, the majority (∼60%) of lower extremity muscle groups decreased their peak sEMG amplitude for all periods (t_1_, t_5_, and t_10_) with no change in the level of muscle activation in the remaining 40% of lower limb muscles. However, in post loaded condition (ST + ES), compared to post unloaded condition (ES-alone), there was a restoration in muscle activation that endured the continuous 10-min stepping. Across 40% of the lower limb muscles, the increased range (20–95%) in peak muscle activation amplitude was in all periods (t_1_, t_5_, and t_10_) especially for the knee extensors (VL, RF) and the ankle flexors and extensors (Figure 2); significantly, post ST + ES loaded condition restored a diminished left VL muscle activation amplitude.

### Load Training Affects Spinal Excitability

After the unloaded training condition (ES-alone), compared to baseline, treadmill stepping evoked increased co-inhibition and lessened muscle amplitude responses for bilateral ankle flexors and extensors. However, these dampened sEMG responses were reversed during the 10-min stepping trial post loaded training condition (ST + ES), compared to ES-alone, for an increase in overall co-excitability (and amplitude) of the plantar flexor/extensor muscle pairs: TA/GN and TA/S (Figure 3).

Moreover, there appears to be a potential adaptation of spinal networks and muscle activation response due to stand training (Beres-Jones and Harkema, 2004; Ichiyama et al., 2008). Stand training, or loading, increased the afferent input (with ES) resulting in increased peak sEMG bursts and burst duration during the stance phase of stepping (Figures 1, 2 and Table 2). Specifically, peak sEMG occurred on peak load (Figure 1) at mid-stance for S, GN, and TA during weight-bearing stepping. Similar temporal sEMG responses for amplitude were seen in AB for GN and S muscles, except TA. TA activation bursts for AB occur at terminal swing and push-off at terminal stance (Figure 1). Similar spinal circuitry adaptations were reflected in left VL muscle activation. In post ES-alone, sEMG activation bursts for VL were diminished during 10-min stepping, only to be restored after longitudinal ST + ES load training (Figure 1).

Lack of weight bearing, or lack of afferent input, and conversely load training, or full weight bearing for afferent input, can have opposing effects on spinal networks and spinal excitability (Beres-Jones and Harkema, 2004; Forrest et al., 2008b). Beres-Jones and Harkema (2004) and Forrest et al. (2008a) established longitudinal, or even single-dose, step-training session resulted in greater sEMG amplitudes in GN, S, and TA during treadmill stepping for individuals with motor-complete injuries.

Our investigation shows a temporal coupling (i.e., phasing) of TA/GN sEMG amplitude during stepping (Figure 1), which is inappropriate for loading on the ipsilateral limb during stepping. The GN/S coupling is a more appropriate one during stepping. Forrest et al. (2008a) reported a similar co-contraction/co-excitation for the GN, S, and TA during loading after a high-dose locomotor training (LT) protocol for motor-complete SCI (Forrest et al., 2008b). After LT, the ankle joint at times would plantarflex on foot landing where the left TA had difficulty to concentrically contract against gravity to promote ankle dorsiflexion (Forrest et al., 2008b). In these experiments, the continued stepping with ankle plantarflexion at floor contact was likely to compromise afferent proprioceptive sensory input at foot floor contact and early stance and could have contributed partially to the co-contraction of the left TA and GN muscles. Beres-Jones and Harkema (2004) also suggested that the increased loading on the contralateral lower limb could have enhanced ipsilateral lower limb muscle activation; therefore, loading may not only facilitate ipsilateral extension but also may simultaneously facilitate contralateral flexion. Ultimately, the co-contraction of the left TA and the left GN needs further investigation (Beres-Jones and Harkema, 2004).

### Trunk Muscle Activation and Center of Mass

At baseline, the ${\hat{C\,o\,M}}_{\text{T}}$ data reflected an overall lack of decoupling for the cervical and thoracic trunk muscles during ipsilateral left and contralateral right leg swing during stepping; the ${\hat{C\,o\,M}}_{T,\mathrm{ML}}$ trajectory projected beyond *BoS* midline (i.e., 50%). The lack of ipsilateral left trunk muscle activation combined with the stronger contralateral superior trunk muscles (SES) determined the overall path of the ${\hat{C\,o\,M}}_{T,\mathrm{ML}}$ at initial left toe-off (Ceccato et al., 2009). During right leg swing, especially at left mid-stance, there is a lack of contralateral trunk muscle bursts; the only activation of the right SES and right IO muscles provide trunk stabilization, resulting in a “pull” of the ${\hat{C\,o\,M}}_{T,\mathrm{ML}}$ further right of the *BoS* midline (Ceccato et al., 2009).

### Lower Limb Multimuscle ES Affects Spinal Excitability

In post unloaded condition (ES-alone), the multimuscle ES of the lower limbs increased the neural control of trunk stability during continuous 10-min stepping. Specifically, during continuous 10-min stepping, the increased activation of the left SES and IES as the primary muscles stabilized the trunk during right swing, especially at left mid-stance (Figure 4). Overall, there was greater control of trunk ${\hat{C\,o\,M}}_{T,\mathrm{ML}}$ for more stabilized trunk ${\hat{C\,o\,M}}_{T,\mathrm{ML}}$ to decouple from leg movement during swing (compared to baseline); ${\hat{C\,o\,M}}_{T,\mathrm{ML}}$, trajectory was similar to AB trajectory.

### Load Training and Lower Limb Multimuscle ES Affect Spinal Excitability

The greatest effect to trunk stability with the least amount of trunk excursion during continuous stepping resulted from the loaded condition combined with multimuscle ES (ST + ES) (Figure 4); trunk ${\hat{C\,o\,M}}_{T,\mathrm{ML}}$ excursions from the midline of *BoS* (50%) to the left (43%; AB: 22%) and right (64%; AB: 64%). Increased trunk stabilization during swing was due to the increase in the trunk neural adaptions during right swing. During right swing, especially at the left mid-stance, primary stabilizers for trunk control, left IES, and IO increased their amplitude and duration, concomitant to the decrease in right trunk muscles (SES, EO, and IO). The decrease in ipsilateral right trunk muscles are important to trunk stability during right swing (especially initial swing); they are known to provide a supportive, secondary role with a specific activation pattern occurring simultaneously with the main primary contralateral trunk stabilizers (Ceccato et al., 2009).

Previously, we have reported (Momeni et al., 2019) that ST + ES training for motor complete SCI increased neuromuscular and postural trunk control during standing, compared to post ES-alone and similar to the AB control during quiet standing. In addition, we reported gains in function that affected activities of daily living: (i) wheelchair transfers required less assistance, (ii) increased functional reach for improved seated balance, and (iii) increased independent seated trunk rotation ability. The current investigation extends previous findings to show that longitudinal stand training with components of stand retraining and stand adaptability can increase independent trunk control even in dynamic treadmill stepping conditions. In the unloaded condition (ES-alone), while there were gains in trunk stabilization during stepping (Figure 4), lower limb muscle activation decreased and co-inhibition increased during the continuous stepping (Figure 3).

With the addition of stand training to multimuscle ES, there was an increased longitudinal afferent input to the spinal network and increased excitability to normalize the trunk stability to resemble able-bodied trunk stability during stepping. Moraud et al. (2018) has suggested that these gains in postural control may also improve stepping quality in the lower extremity via the optimal medial–lateral trunk ${\hat{C\,o\,M}}_{\text{T}}$ excursion assisting in preserving the muscle spindle feedback to the lower agonist/antagonist muscles in the lower limb during stepping (Moraud et al., 2018).

After the combined ST + ES training, lower limb muscle amplitude increased to enhance treadmill stepping for a continuous 10-min stepping trial (Figure 5). However, the increased co-excitation of the lower limb muscles (GN/TA) reflected an inappropriate intralimb stepping pattern. The observed inter-limb muscle activation spatial–temporal patterns and the spatial–temporal pattern of the α-motorneuron pool in the lumbosacral segment, especially the upper lumbar segment, for the SCI participant, are not fully representative of the AB’s lumbosacral segment during terminal stance and terminal swing phases of stepping (Figure 5; Ivanenko et al., 2006); this is possibly because the stand training intervention was directed toward independent standing and not stepping. Using the α-motorneuron activity maps for this individual provides an approximate location of these motorneuron pools in the spinal cord (Yakovenko et al., 2002; Ivanenko et al., 2006). The maps, based on EMG profiles, do not directly represent individual contributions of muscles, but rather, they represent the organization of the spinal network and its motor output during stepping for this individual after the completion of the ES-alone and ST + ES interventions.

This preliminary case study is limited by the sample size of one, which makes it infeasible to obtain statistical significance in order to generalize its findings to the larger population. Future work needs to evaluate the intervention with a greater sample size for the effect of the intervention paradigm on trunk and lower limb gains. Another limitation of this study was the consecutive order of the interventions, without a washout period. Although this study focuses on the sequential changes and compares ST + ES only to ES-alone, a washout period would be preferable in future studies to avoid potential carryover effects comparing multiple interventions. Moreover, the therapists who performed the assisted stepping during data collection were not blinded to the type of intervention.

Overall, this study has shown, for one individual with SCI, an increase in dynamic postural stability and neural gains for integrated trunk and lower extremities after a dynamic standing intervention compared to an unloaded supine intervention for the same multimuscle ES protocol. The spinal neural networks and biomechanical adaptations reflected an increase in afferent input and spinal excitability (Edgerton and Roy, 2009) during training and the possible interactive neural control of trunk circuits and lower limb during locomotion as described by Moraud et al. (2018). Further research should evaluate the influence of the proprioception, mediolateral trunk orientation, and trunk and lower limb motor pools on the neural and mechanical intersegmental relationship between trunk and lower extremity during locomotion, as well as how all these constraints influence or modulate the overall postural recovery during standing and locomotion.

## Data Availability Statement

The raw data supporting the conclusions of this article will be made available by the authors, without undue reservation, to any qualified researcher.

## Ethics Statement

The studies involving human participants were reviewed and approved by the Kessler Foundation Institutional Review Board. The patients/participants provided their written informed consent to participate in this study.

## Author Contributions

GF and EG contributed to the conception and design of the study. KM and AR organized the database. KM performed the statistical analysis and wrote the first draft of the manuscript. GF and AR wrote sections of the manuscript. All authors contributed to manuscript revision, and read and approved the submitted version.

## Conflict of Interest

The authors declare that the research was conducted in the absence of any commercial or financial relationships that could be construed as a potential conflict of interest.
